# Multifocal acoustic radiation force-based reverberant optical coherence elastography for evaluation of ocular globe biomechanical properties

**DOI:** 10.1117/1.JBO.28.9.095001

**Published:** 2023-09-11

**Authors:** Taye Mekonnen, Christian Zevallos-Delgado, Manmohan Singh, Salavat R. Aglyamov, Kirill V. Larin

**Affiliations:** a University of Houston, Department of Biomedical Engineering Houston, Texas, United States; b University of Houston, Department of Mechanical Engineering, Houston, Texas, United States

**Keywords:** optical coherence elastography, biomechanical properties, eye globe, multifocal acoustic radiation force, reverberant shear wave, elastography

## Abstract

**Significance:**

Quantifying the biomechanical properties of the whole eye globe can provide a comprehensive understanding of the interactions among interconnected ocular components during dynamic physiological processes. By doing so, clinicians and researchers can gain valuable insights into the mechanisms underlying ocular diseases, such as glaucoma, and design interventions tailored to each patient’s unique needs.

**Aim:**

The aim of this study was to evaluate the feasibility and effectiveness of a multifocal acoustic radiation force (ARF) based reverberant optical coherence elastography (RevOCE) technique for quantifying shear wave speeds in different ocular components simultaneously.

**Approach:**

We implemented a multifocal ARF technique to generate reverberant shear wave fields, which were then detected using phase-sensitive optical coherence tomography. A 3D-printed acoustic lens array was employed to manipulate a collimated ARF beam generated by an ultrasound transducer, producing multiple focused ARF beams on mouse eye globes *ex vivo*. RevOCE measurements were conducted using an excitation pulse train consisting of 10 cycles at 3 kHz, followed by data processing to produce a volumetric map of the shear wave speed.

**Results:**

The results show that the system can successfully generate reverberant shear wave fields in the eye globe, allowing for simultaneous estimation of shear wave speeds in various ocular components, including cornea, iris, lens, sclera, and retina. A comparative analysis revealed notable differences in wave speeds between different parts of the eye, for example, between the apical region of the cornea and the pupillary zone of the iris (p=0.003). Moreover, the study also revealed regional variations in the biomechanical properties of ocular components as evidenced by greater wave speeds near the apex of the cornea compared to its periphery.

**Conclusions:**

The study demonstrated the effectiveness of RevOCE based on a non-invasive multifocal ARF for assessing the biomechanical properties of the whole eyeball. The findings indicate the potential to provide a comprehensive understanding of the mechanical behavior of the whole eye, which could lead to improved diagnosis and treatment of ocular diseases.

## 1 Introduction

Knowledge of the biomechanical properties of the various interconnected ocular components is critical for understanding the physiology of the eye globe, detecting ocular diseases, and designing effective treatments and surgical procedures. For example, measuring the biomechanical properties of the cornea and sclera can help detect the early signs of glaucoma, a condition that can be characterized by increased intraocular pressure (IOP).^1^ By understanding how different ocular components work together to resist changes in IOP, clinicians can assess glaucoma risk and develop effective treatment strategies for managing glaucoma. Furthermore, biomechanical models of the eye can be used to design personalized therapeutic procedures for implanting intraocular lenses or performing corneal transplant surgeries, ensuring that the procedures are safe and effective.

Recent advances in innovative techniques for measuring the biomechanical properties of individual ocular tissues hold great promise for improving our understanding of ocular biomechanical properties. For example, the ocular response analyzer,^2^^,^^3^ CorVis,^4^ Brillouin microscopy,^5^^,^^6^ and optical coherence elastography (OCE)^7^ have shown potential for quantifying the biomechanical properties of individual ocular tissues with high precision and accuracy. However, there is still a gap in the methods used for detecting the biomechanical properties of various ocular components simultaneously. While it is important to understand the biomechanical properties of individual ocular tissues, the whole eye globe is a complex and interconnected system. The interactions between different ocular components play a crucial role in determining the overall ocular functions.^1^ For instance, previous research utilizing finite element analysis has demonstrated that the lens, iris, and other ocular muscles contribute significantly to the mechanical aspects of corneal deformation.^8^ Therefore, it is essential to determine the mechanical properties of the interrelated parts, such as the cornea, lens, sclera, iris, and retina, for an accurate understanding of the eye’s mechanical behavior.

Several studies have demonstrated promising outcomes in estimating the biomechanical properties of multiple ocular components simultaneously. One such study involved measuring displacement in response to eye globe inflation using multiple cameras and a laser displacement sensor to enable the assessment of regional variations in the stiffness of the cornea and sclera.^9^ However, this study was limited to the surface of the eye globe, and internal parts, such as the lens and iris, were not assessed. On the other hand, high-field magnetic resonance imaging has been used to assess the displacement of the whole eyeball with a reasonable resolution, but its clinical application is limited due to long scan times and motion artifacts.^1^ Ultrasound elastography (USE) is another promising method and has been used to determine the biomechanical properties of the sclera and optic nerve simultaneously.^10^ A recent study presented the first comprehensive elasticity assessment of the whole eyeball using USE, characterizing the biomechanical properties of the cornea, lens, iris, optic nerve hypoplasia, and peripapillary sclera.^11^ However, this method involved a mechanical shaker to induce deformation in the tissues, which may cause discomfort during *in vivo* application and hence may influence measurement results. Additionally, the resolution of USE is relatively low.

OCE^12^^,^^13^ has emerged as a high-resolution technique to quantify the biomechanical properties of multiple ocular components simultaneously. For example, OCE has been used to study the biomechanical properties of the anterior eye (cornea, limbus, and anterior sclera simultaneously),^14^ the lens and cornea,^15^ and the cornea and retina.^16^ Although these studies provided valuable insights into the biomechanical properties of different ocular components, measurements were limited to only a few ocular components at a time due to the limited tissue penetration depth. The recent introduction of reverberant OCE (RevOCE), which assumes the existence of complex three-dimensional reverberant shear wave fields in the tissue,^17^ aims to overcome the above limitations.^18^ However, conventional mechanical shakers employed in existing RevOCE techniques that directly contact tissues may not be suitable for assessing sensitive (delicate) ocular tissues.

In this context, the aim of the current study is to demonstrate the capability of a non-invasive RevOCE in assessing the biomechanical properties of the entire eye globe with high mechanical resolution and contrast. We present a multifocal acoustic radiation force (ARF) system, which was implemented using an array of acoustic lenses and a single-element ultrasound transducer as an excitation source to induce reverberant shear wave field in mouse eyeballs.^19^ The induced reverberant shear wave field was then imaged with phase-sensitive optical coherence tomography (PhS-OCT) to obtain a volumetric wave speed map of the entire eye globes. Shear wave speeds were quantified in different ocular components, including the cornea, iris, sclera, lens, and retina, yielding a comprehensive understanding of not only the relative stiffness variations among these interconnected parts but also the regional heterogeneity within each ocular component (e.g., cornea).

## 2 Materials and Methods

### 2.1 Mouse Eyeball Samples

Experiments were conducted on three freshly exercised mouse eyeballs (within 2 h after euthanasia). The eyeballs were cleaned of extraocular tissues, maintained in 1 X phosphate-buffered saline (PBS) solution, and frequently hydrated during imaging. The average IOP of the eyeballs, as measured by a rebound tonometer (Tonolab, iCare Finland Oy, Finland) before the start of OCE experiments, was ∼8.8  mmHg; thus, no IOP control was implemented.

### 2.2 Systems and Data Acquisition

**Fig. 1:**
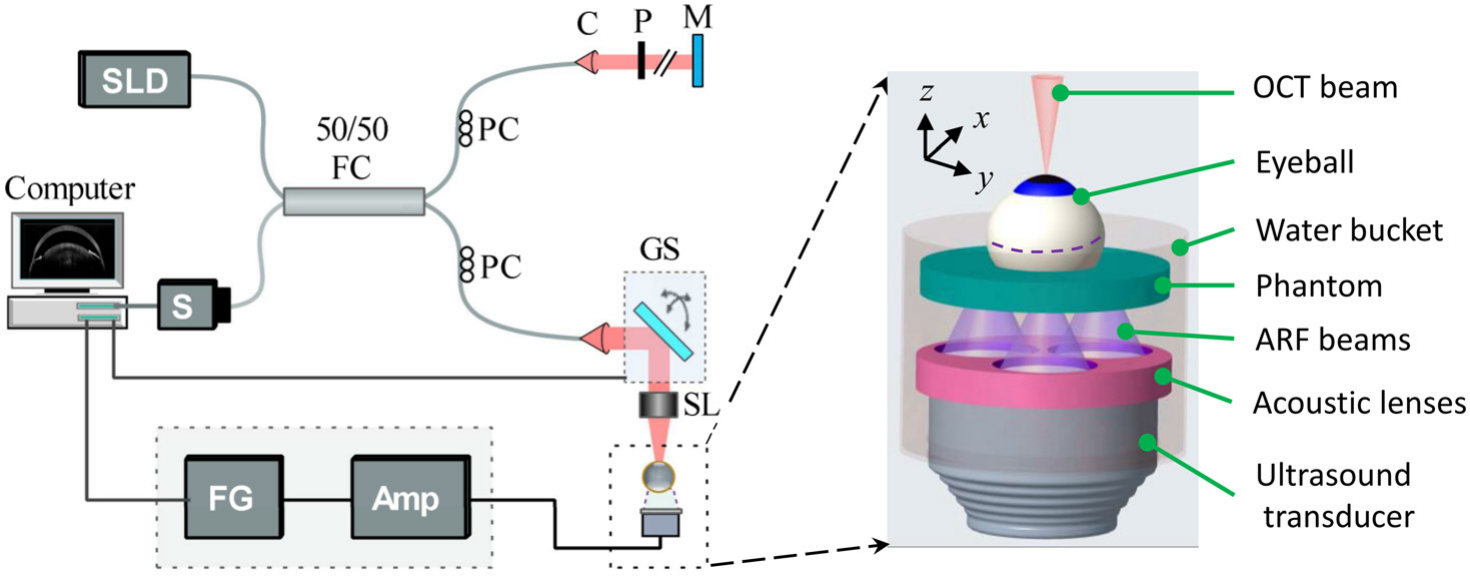
Schematic of the RevOCE system consisted of an ultrasound transducer coupled with array of acoustic lenses to produce multifocal ARF and a PhS-SDOCT system for imaging elastic wave propagation induced by the ARF. Amp, RF amplifier; C, collimator; FC, fiber coupler; FG, function generator; GS, 2D galvo scanner; M, reference mirror; P, pinhole; PC, polarization controller; S, spectrometer; SL, scan lens; and SLD, a superluminescent diode. The inset to the right is a 3D view of the acoustic lens and transducer system showing the multifocal ARF beams used to induce reverberant wave fields in mouse eyeballs that were mounted on a gelatin phantom (2% w/w concentration). The ARF beams were focused near the eyeball equator, aligned along the dashed lines surrounding its perimeter and positioned ∼120  deg apart from each other around the circumference. 3D OCE data were acquired by the 2D galvo scanner motion in the x-y plane. The sample setup was translated manually along the z-axis to repeat the 3D OCE measurement at the posterior of the eyeball.

As shown in Fig. 1, the RevOCE system consisted of a lens-transducer system to generate multifocal ARF ^19^ and a PhS-OCT system to detect the resulting vibrations in the ocular tissues. A collimated ARF beam was produced by a 13 mm diameter single-element, 3.5 MHz ultrasound transducer (C382-SU, Olympus Co., Japan) and was coupled to an acoustic lens array composed of three plano-concave lenses. The individual lens geometries were specifically designed with a focal length of 9.7 mm, an aperture diameter of 6.6 mm, and a working distance of 6.4 mm. The lens array, which was 3D-printed using rubber-like material (TangoGray FLX950, Stratasys, Israel), was designed to produce three focused ARF beams with a spatial separation of ∼2.65  mm in the focal plane. Measurements with a hydrophone of 0.2 mm sensor diameter (NH0200, Precision Acoustics Ltd, United Kingdom) confirmed the presence of the three focal spots separated by ∼2.65  mm. The spot size, defined as the full width half maximum of the normalized pressure intensity at the focal plane, was determined to be 1.13 mm. The induced reverberant shear wave field was detected using a PhS-OCT system characterized by displacement stability, axial resolution in air, and transverse resolution of 0.28 nm, ∼9  μm, and ∼8  μm, respectively.^20^ The OCT system was operated at an A-line rate of 25 kHz. In prior research, we used seven foci ARF to generate reverberant shear wave fields.^21^ Despite utilizing only three foci excitation in the current study to accommodate the size of a mouse eyeball, our preliminary findings and prior studies^22^ confirm that using three or more sources can effectively generate reverberant shear wave fields in tissues.

During OCE imaging, the eyeball was mounted on a soft gelatin phantom (inset in Fig. 1, 2% w/w concentration), which had very low acoustic attenuation. To induce reverberant shear wave fields in the mouse eyeball, an arbitrary function generator (DG4162, RIGOL Tech, China) produced a 3.5 MHz sinusoidal signal modulated by ten cycles of a 3 kHz rectangular tone burst, which was then amplified by a power amplifier (1040L, Electronics & Innovation, Ltd., United States) feeding the transducer. Our selection of 3 kHz was a careful compromise between generating shorter excitation wavelengths necessary for inducing multiple internal reflections and avoiding excessive attenuation of waves. The excitation was synchronized with the OCT system frame trigger during OCT M-C mode scans, a technique utilized to obtain four-dimensional (4D) data in OCE, with “M” representing motion (temporal data) and “C” denoting volumetric (3D spatial data). This method involves capturing multiple A-scans at the same transverse location over time through M-mode scanning, which is repeated at every lateral location using a raster scanning procedure. In this study, the scan acquired 151×151 points over a lateral region of interest of 3.8 mm by 4.2 mm, with each M-mode scan consisting of 400 A-lines. As the OCT system axial imaging range was limited to ∼2.5  mm, we scanned the entire eyeball using a two-step imaging process. First, we adjusted the OCT imaging head position to enable RevOCE measurements on the anterior eye. Next, the imaging head was axially translated to focus on the posterior eye, and the RevOCE measurements were repeated.

**Fig. 2:**
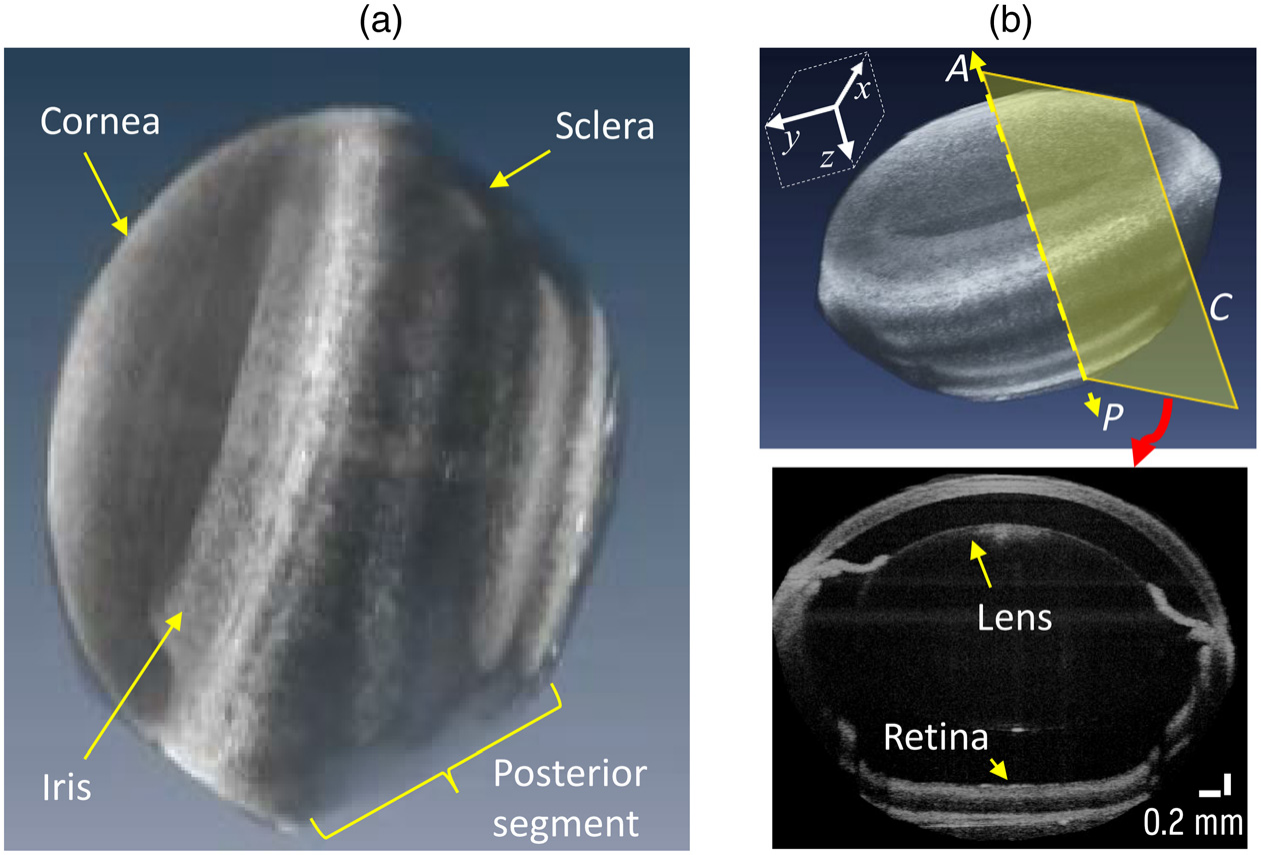
(a) A 3D OCT scan reconstruction of a mouse eyeball, illustrating its representative gross morphology. (b) Top: the volumetric image of the eyeball with the z-axis indicating the anterior-posterior (A–P) direction which is along the optical axis of the OCT objective beam and eyeball optical axis. Bottom: a cross-sectional slice taken in plane C.

### 2.3 Data Processing and Analysis

The 4D OCE data (3D spatial and 1D temporal) from the two-step measurement were processed to reconstruct a 3D map of the elastic wave speed in the whole eyeball using a custom MATLAB^®^ R2021a (The MathWorks, Inc., United States) program. First, the phase shift, ΔØ, between successive A-scans was used to compute the axial particle velocity, $v_{z}=\lambda_{o}\Delta Ø/(4\pi n\Delta \tau )$, where $\lambda_{o}=840\;\mathrm{nm}$ was the center wavelength of the OCT light source, n=1.46 was the refractive index of the ocular tissues, and Δτ=40  μs was the time interval between successive A-lines. The particle velocity volumetric frames were denoised using both temporal and spatial frequency filters. In the temporal domain, an impulse response filter centered around the excitation frequency of 3 kHz and full width half maximum bandwidth of 0.1 kHz, which was selected after some pre-analysis, was used to maintain signal integrity while minimizing noise. Similarly, a 2D spatial bandpass filter was applied to remove unwanted motion noise. The wavenumber filter was designed by setting the cutoff frequencies based on previously established ranges of shear wave speed in soft tissues, such as the mouse eye, which typically falls between 0.4 and 9.5  m/s.^20^^,^^23^ Then, the lower $\left(k_{l}\right)$ and upper $\left(k_{u}\right)$ limits of the wavenumber filter were determined using the relation $\left[k_{l},k_{u}]=[2\pi f/v_{u},2\pi f/v_{l}\right]$, where f=3  kHz was the excitation frequency and $v_{l}=0.4\;m/s$ and $v_{u}=9.5\;m/s$ were the assumed speed limits. After denoising, the local shear wave speed, $v_{s}$, was estimated using the relation $v_{s}=2\pi f/k$, where k was the local wavenumber obtained by undertaking a 2D autocorrelation (window size = 0.4 mm by 0.4 mm) of the particle velocity volume followed by fitting the autocorrelation profiles to the analytical solutions of the reverberant shear wave field model.^17^^,^^18^^,^^24^^,^^25^ The window size was selected to be equal to at least half of the wavelength of the induced elastic wave to achieve a better estimate of the wave speed and not compromise the achievable elastic resolution. The described procedure was implemented on the particle velocity reverberant frames at all depths, encompassing all *en-face* frames of each 3D particle velocity volume. Wave speed results in different regions of the eye were compared using Student’s t-test.

## 3 Results

### 3.1 Structural OCT Images of the Eye Globe

The structural image of the whole eyeball was reconstructed from the M-C OCT scan. Figure 2 shows a typical structural image of mouse eyeball depicting both the anterior and posterior segments. The volume render in Fig. 2(a) clearly reveals the anterior segment with the cornea, iris, lens, and anterior region of the sclera. The major ocular components can be better revealed using a cross-sectional view indicated Fig. 2(b) (bottom), which was extracted from the yellow sectional plane indicated in Fig. 2(b) (top). Here, all the major ocular components from anterior (i.e., cornea, lens, iris, cornea, and anterior segment of sclera) to posterior (i.e., retina, choroid, and posterior scleral segment) are discernable. The ability of the OCT system to discern the ocular components is critical to correctly relate the elasticity differences measured by RevOCE.

### 3.2 Wave Propagation and Speed Maps

**Fig. 3:**
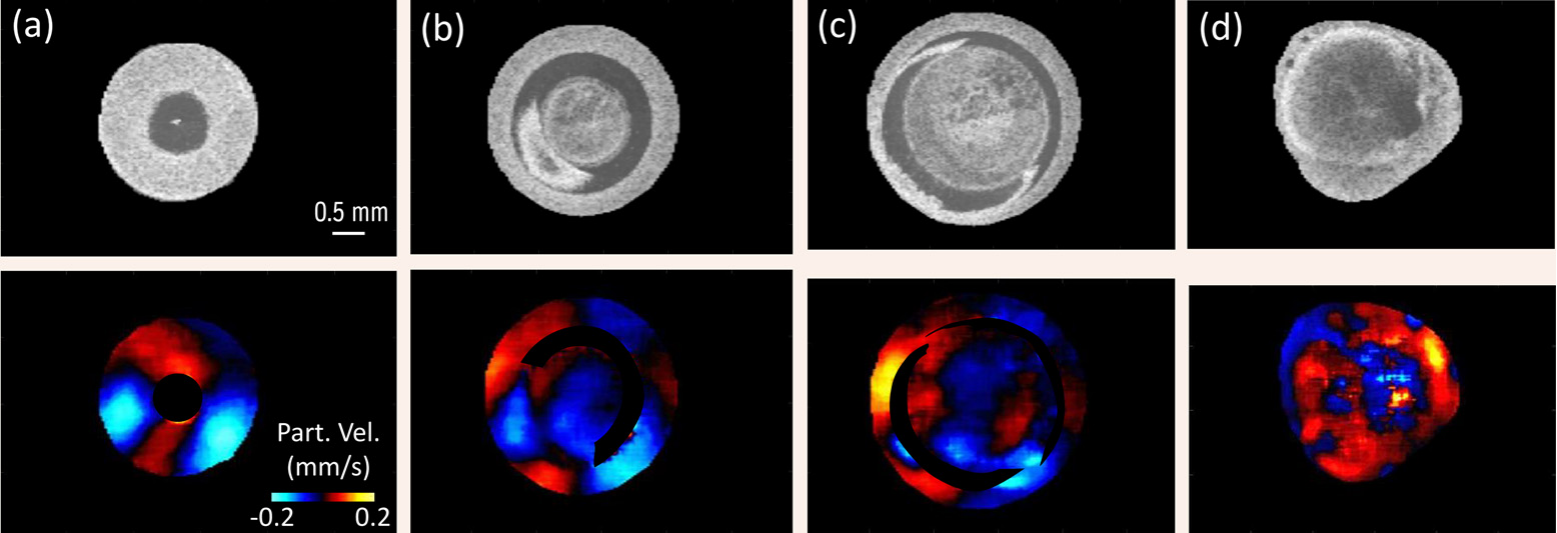
Elastic wave propagation snapshots at t=2.12  ms after excitation at different depths in *en-face* slices of the eyeball showing (a) cornea (near apex); (b) cornea, iris, and lens; (c) cornea, iris, lens, and sclera (partial view); and (d) posterior (across retina). Clearly, the reverberant shear wave field is generated in all regions of the eyeball from the apex of cornea to the posterior segment of the eye.

The reverberant shear wave field induced in the eye globe can be visualized by taking the instantaneous particle velocity map in various *en-face* images positioned in parallel planes orthogonal to the anterior-posterior pole. Figure 3 depicts representative *en-face* OCT images (top row) taken from different *en-face* positions and instantaneous wave motion snapshots (bottom row) at t=2.12  ms after start of the excitation. Figure 3(a) shows the cornea region near the apex while Fig. 3(b) shows a partial view of the lens and iris in addition to the cornea. In Fig. 3(c), a partial view of the sclera as well as the cornea, lens and iris are visible. Finally, the last column, Fig. 3(d), shows a slice in the posterior segment near the retina. Evidently, the reverberant shear wave field was well-established throughout the whole eye globe (Fig. 3, bottom row).

**Fig. 4:**
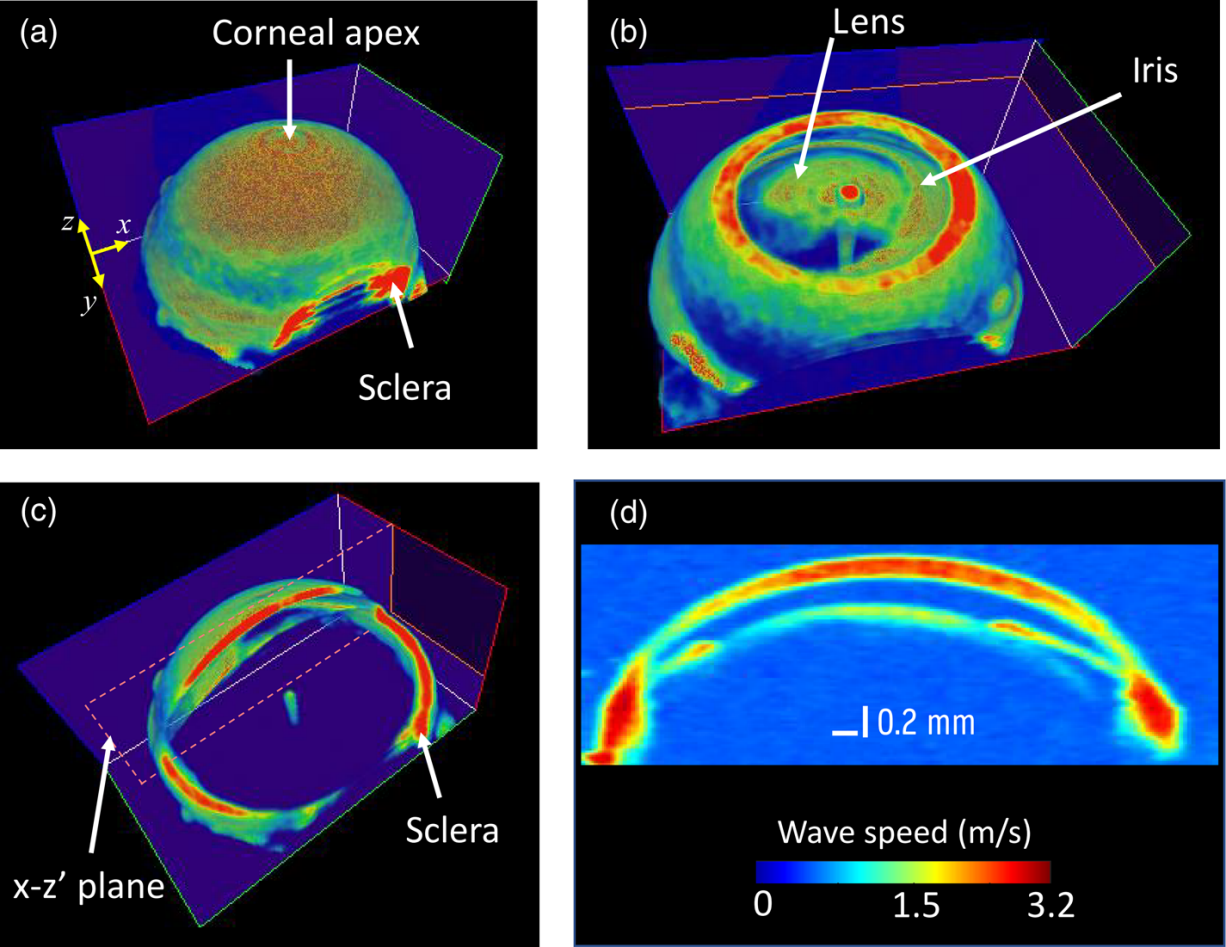
(a) A typical 3D speed map of anterior eye segment with slight cut plane in x-z plane to show the sclera. (b) An enface cut in the x-y plane revealing the lens, cornea and iris regions. (c) Partial planar cuts showing the depth and enface regions (sclera). (d) A cross-sectional slice taken from the $x\text{-}z^{\prime }$ plane in panel (c) depicting elastic wave speed differences in the main components in the anterior segment.

Figure 4 depicts a wave speed map in the anterior segment of the eyeball. This map allows us to identify the major components of the anterior eyeball, as seen in Figs. 4(a) and 4(b). The cross-sectional images shown in Figs. 4(c) and 4(d) reveal that there is a noticeable difference in the elastic wave speed among these components. Nevertheless, it is worth noting that the wave speeds at the air-tissue interfaces (e.g., cornea exterior surface) and the liquid-tissue interfaces (e.g., lens and iris anterior surface) seem to exhibit similar trends across all ocular components, with consistently lower values compared to the wave speed within their inner regions as shown in Fig. 4(d). This is mainly attributed to the correlation window in which tissue and non-tissue pixels are included in these regions. To avoid this issue, it is possible to segment the eyeball and only analyze the tissue regions using autocorrelation-based speed analysis. Nonetheless, one can still identify differences in the elastic wave speed by examining the cross-sectional view, i.e., x-z plane shown in Fig. 4(c), which is displayed in Fig. 4(d). The annotated regions display a significant variation in elastic wave propagation speed, with the lens and iris exhibiting a slower wave speed compared to the cornea and sclera.

### 3.3 Comparison of Wave Speeds in Different Ocular Components

**Fig. 5:**
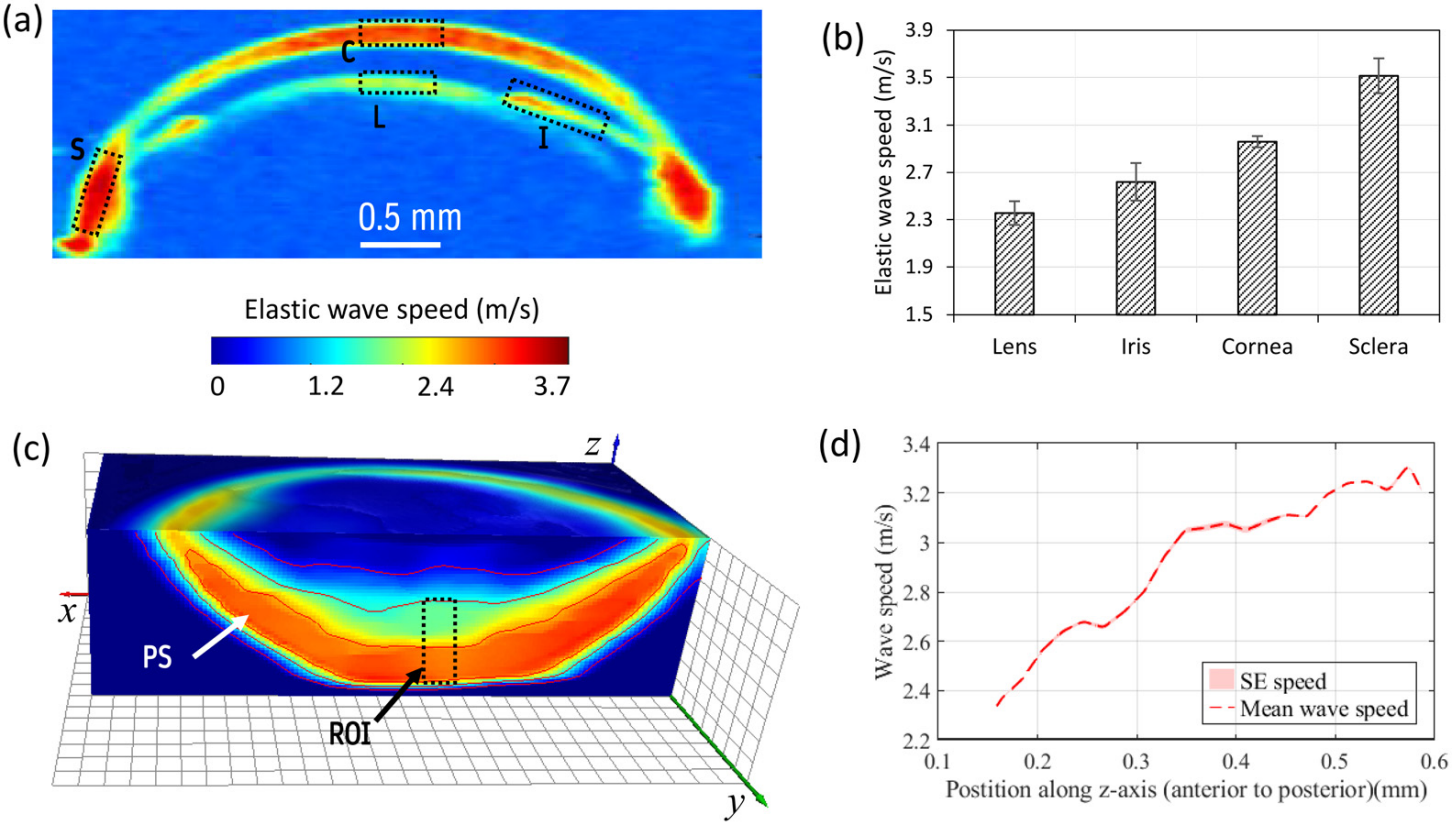
Quantifying the elastic wave speeds in different ocular components. (a) Wave speed map in a representative anterior eye cross-section. Regions of interest for quantitative estimation of the wave speeds in each ocular component are indicated by the dashed rectangular regions; C, cornea; I, iris; L, lens; and S, anterior sclera. (b) Comparison of wave speeds of ocular components in the anterior eye. The mean wave speeds were quantified from the dashed rectangular regions in panel (a) excluding any fluid (N=3 eyes). (c) Partial view of a 3D wave speed map in the posterior segment of the eyeball. PS: posterior sclera. (d) Elastic wave speed profile along the anterior-posterior direction for the dashed rectangular region shown in panel (c) and annotated as ROI indicating increase in wave speed from the inner layer (i.e., retina) to the outer layer (i.e., posterior sclera).

Figure 5 demonstrates the relative differences in wave speed measured in the anterior segment, including the cornea, lens, iris, and anterior sclera. Specific regions within each component were selected, and the means of their speed differences were plotted in a bar graph. As indicated in Fig. 5(b), the anterior sclera exhibits larger wave speed compared to the components in the anterior segment. However, it is essential to note that the corneal wave speed is not uniform, potentially indicating regional differences in corneal stiffness. The next section will detail a comparison of regional variations in corneal wave speed.

Similarly, the wave speed map in the posterior segment shown in Fig. 5(c) indicates relative variations in the sclera, choroidal and retina regions. Figure 5(c) displays a partial view of a volumetric wave speed map of the posterior segment, showing variations across the thickness of the eyeball. By selecting differential rectangular regions indicated by the dashed rectangle annotated as ROI in Fig. 5(c), we quantified the speed variations along the posterior eyeball. Figure 5(d) presents the anterior-posterior profile of the wave speed in the dashed rectangular region, revealing a change in wave speed from approximately 2.4 to 3.2  m/s in the anterior (retina) to the posterior (sclera) regions, respectively. Moreover, the bumps in the profile between the extreme ends potentially indicate the presence of different layers (e.g., retina layers or the choroidal region). This information provides further insight into the differences in elasticity among the different layers of the posterior segment.

### 3.4 Regional Variation of Wave Speed in the Cornea

**Fig. 6:**
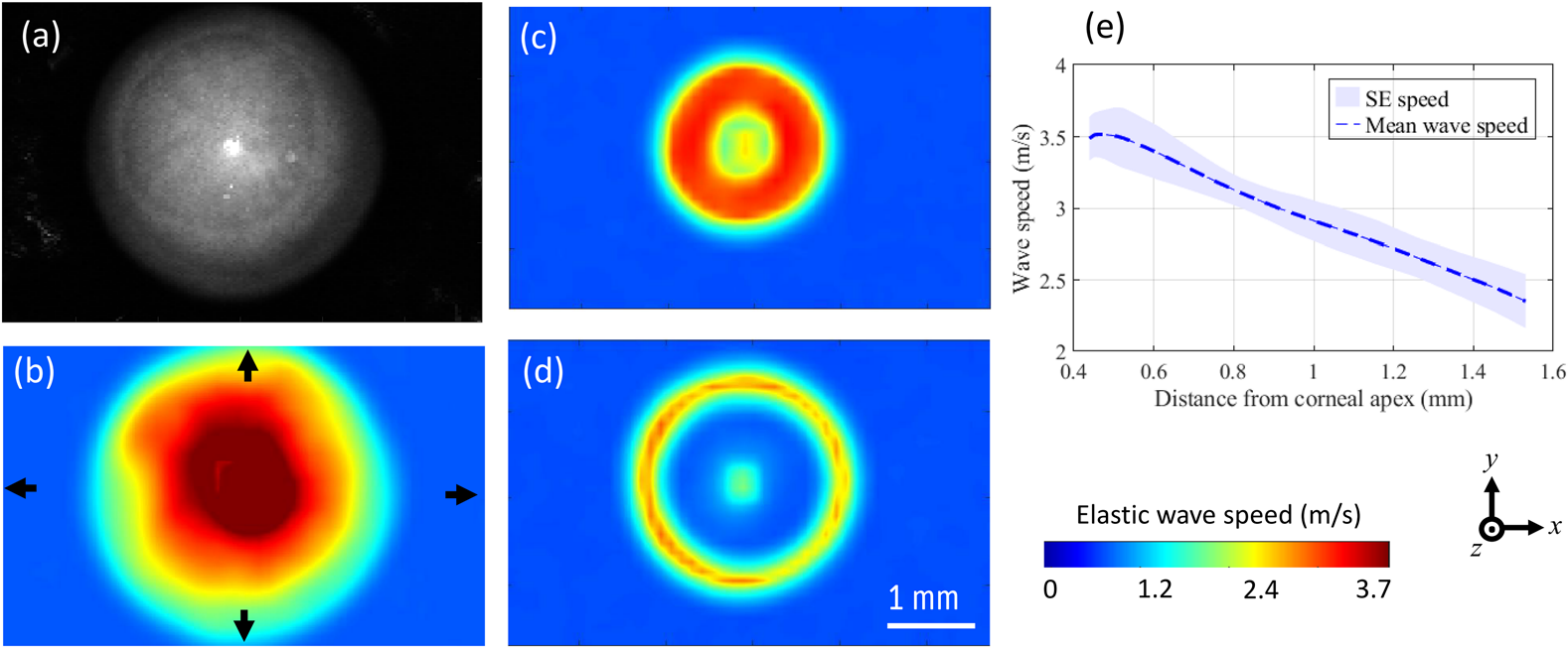
Regional variations of elastic wave speed in the cornea. (a) *En-face* structural image of representative mouse cornea. (b) Depth averaged *en-face* elastic wave speed map in the cornea in panel (a). Representative elastic wave speed maps in the *en-face* of cornea taken at (c) 0.19 mm and (d) 0.36 mm posterior to corneal apex. (e) Radial wave speed profile along the four arrows shown in panel (b), i.e., from the apex to the periphery. SE: standard error of the four speed profiles taken along the directions shown by the arrows in panel (b).

The ability to quantify the biomechanical properties of the entire eye globe offers a significant advantage by allowing for the evaluation of the localized stiffness of specific ocular components such as the cornea. In Fig. 6, the variation in elastic wave speed of the cornea is illustrated. The depth-averaged (i.e., along the optical axis of the eye) structural image of the cornea is shown in Fig. 6(a), along with its corresponding wave speed map in Fig. 6(b). The wave speed map reveals that the highest elastic wave speed is observed at the corneal apex, with the speed decreasing progressively towards the pre-limbal region. A clearer representation of this regional variation can be obtained by referring to the two *en-face* speed maps displayed in Figs. 6(c) and 6(d), which were obtained from two parallel planes separated by 0.19 mm along the anterior-posterior pole. The speed profile from the apex to the periphery, an average of the profiles along the four paths represented by the arrows in Fig. 6(d), is shown in Fig. 6(e) and demonstrates a noticeable reduction from the apex to the pre-limbal region, decreasing from ∼3.50 to ∼2.35  m/s over a radial distance of 0.75 mm. Furthermore, the radial non-uniformity of the speed map in Fig. 6(a) is of interest since it potentially suggests that the stiffness of different corneal quadrants is heterogeneous. However, it is not possible to identify which regions are inferior-superior or temporal-nasal since the experiments did not note the anatomical alignment of the eyeball.

## 4 Discussion and Conclusion

The purpose of this study was to explore the potential of a novel RevOCE technique for quantifying the biomechanical properties of the whole eyeball. A multifocal ARF method was utilized to induce reverberant shear wave fields, which were then imaged using PhS-OCT. The results show that the technique can be used to quantify the biomechanical properties of the whole eyeball, providing insight into the relative differences in the stiffness of different ocular components of the intact whole eyeball. For the experimental conditions of the current study, the wave speed was found to be the greatest in the sclera, approaching 3.7  m/s in its anterior region. The mean elastic wave speed in the apical cornea region was found to be significantly higher when compared to the mean elastic wave speed in the apical region of the lens (p<0.005) and the pupillary zone of the iris (p=0.003) as indicated by a Student’s t-test. Likewise, the scleral elastic wave speed exhibited significantly higher value compared to that of the cornea, iris, and lens (p<0.001). Additionally, the iris showed a relatively higher wave speed compared to the lens (p=0.014). Although the posterior sclera had a relatively lower speed compared to the anterior sclera, this difference was not found to be statistically significant (p>0.05). If the IOP were maintained at a higher level (≥12  mmHg), it is possible that the contrast in wave speed between the cornea, lens, and iris would have been even more pronounced.^11^^,^^14^^,^^26^^,^^27^ The elastic wave speeds in the various components of the eye fall within a reasonable range as reported in previous studies.^11^^,^^15^^,^^26^^,^^28^ However, it is worth noting that these values may significantly differ depending on factors such as the IOP, boundary conditions, species, and age of the animal model.^15^^,^^28^^–^^30^

The technique also allowed for the evaluation of localized elastic wave speed within specific ocular components. For instance, the cornea showed regional variation in elastic wave speed, with higher elastic wave speed observed in the central regions and lower speed towards the peripheral regions. This finding is consistent with prior studies,^9^^,^^31^ which has shown increased corneal compliance towards the limbal junction and minimal changes in central corneal curvature in response to IOP variations, suggesting that the central cornea is stiffer compared to the peripheral cornea. The observed variation in deformation across different regions is thought to be influenced by structural factors, including collagen distribution and orientation, as well as the distribution and concentration of elastin fibers in the cornea.^9^ These findings hold significant implications for understanding corneal biomechanics, the pathogenesis of corneal diseases, and the potential for tailored therapeutic approaches.^32^^,^^33^ Moreover, the apparent radial non-uniformity in the elastic wave speed map of the cornea, as shown in Fig. 6(b), particularly at the peripheral regions (i.e., the pre-limbal region), may suggest the existence of potential stiffness variation in the different corneal quadrants. For example, a previous study reported meridional variability in corneal deformation, showing greater deformation in the peripheral cornea at the superior and inferior poles compared to the nasal-temporal region.^9^ This information is essential to establish the correlation between regional biomechanical properties and the underlying collagen fibril structure in each region. Hence, this technique holds great promise in accurately diagnosing corneal diseases that exhibit regional biomechanical heterogeneity, such as keratoconus.^34^^,^^35^ However, it is crucial to note that further studies with a larger sample size and different species (e.g., human) are necessary to confirm this correlation and associate biomechanical heterogeneity with the quadrants of the cornea. Such studies can aid in the development of effective treatment strategies for corneal disorders.^36^^,^^37^

The elastic wave speed profile in the posterior segment in proximity to the optic nerve revealed a gradual increase from the inferior (∼2.4  m/s) to posterior (∼3.4  m/s) direction, with discernible bumps potentially indicating the presence of biomechanically distinct layers, such as the layers of the retina, the choroidal membrane, and the posterior sclera. Due to the relatively smaller thickness of the layers in the mouse ocular components (tens of micrometers) in comparison to the elastic resolution, it was challenging to visualize each layer of the retina and choroidal membrane accurately.^38^ Therefore, conducting further research on different species with eyeball sizes comparable to humans and with improved elastic resolution would offer a better understanding of the capabilities of RevOCE in identifying various layers of the posterior segment of the eyeball. However, previous work has shown that RevOCE has improved mechanical contrast as compared to traditional wave-based OCE,^18^ so further studies with higher excitation frequencies are underway.

While this study successfully determined the elastic wave speed of various ocular tissues using OCE, there are some limitations that could be addressed in future work. First, the current OCT system has a limited axial imaging range, which necessitates a two-step measurement process. To overcome this limitation, future work may utilize longer-range OCT systems like swept source OCT systems,^39^ enabling faster imaging without the need for adjustment of the axial position of the imaging probe. Second, the excitation orientation and the OCT objective lens were restricted to opposite sides of the eyeball, making it unsuitable for *in vivo* conditions. Future work will consider allowing excitation orientation on the same side of the objective lens to enable *in vivo* assessment, such as with an imaging hole as demonstrated with ring-shaped transducers.^40^^,^^41^ Third, the use of different excitation frequencies could provide valuable viscoelastic properties of the various ocular tissues.

Generally, the RevOCE technique employed in this study offers an unprecedented opportunity to evaluate the biomechanical properties of various ocular components simultaneously. This technique stands out for its superior elasticity resolution when compared to the USE previously reported^11^ and superior mechanical contrast compared to traditional wave-based OCE.^18^ Furthermore, unlike conventional RevOCE methods that utilize mechanical shakers that physically contact tissues directly, which can potentially compromise the integrity of delicate tissues while inducing a reverberant field,^18^^,^^42^^,^^43^ the presented technique relies on ARF, which does not necessitate direct contact between the actuator and the sample, making it more convenient for *in vivo* applications. By providing a more complete understanding of the interrelationship of the biomechanical properties of the different ocular components, this technique can have important implications for both research and clinical practice. For instance, it can help in analyzing the effect of a particular disease on different ocular components, which can lead to more effective diagnosis and treatment strategies that may have been unavailable due to focusing on a specific ocular component only. Furthermore, the ability to concurrently evaluate multiple ocular components is especially useful in monitoring disease progression, where the effect reflected on secondary ocular component due to a diseased ocular component would carry significant value, such as improved diagnostic efficacy. It can also be used to assess the efficacy and side effects of treatment strategies, such as corneal cross-linking for keratoconus,^44^^,^^45^ by monitoring changes in the elastic properties of the cornea as well as other adjacent ocular components, respectively.

## Code, Data, and Materials Availability

All data in support of the findings of this paper are available from the authors upon reasonable request.
